# The moderating effect of physical activity in the relationship between sleep quality and BMI in adults with overweight and obesity

**DOI:** 10.3389/fspor.2025.1455731

**Published:** 2025-03-13

**Authors:** Letizia Galasso, Ramona De Amicis, Lucia Castelli, Andrea Ciorciari, Antonino Mulè, Alberto Battezzati, Simona Bertoli, Andrea Foppiani, Alessandro Leone, Fabio Esposito, Angela Montaruli, Eliana Roveda

**Affiliations:** ^1^ Department of Biomedical Sciences for Health, University of Milan, Milan, Italy; ^2^ International Center for the Assessment of Nutritional Status and the Development of Dietary Intervention Strategies (ICANS-DIS), Department of Food, Environmental and Nutritional Sciences (DeFENS), University of Milan, Milan, Italy; ^3^ Department of Endocrine and Metabolic Diseases, IRCCS Istituto Auxologico Italiano, Obesity Unit and Laboratory of Nutrition and Obesity Research, Milan, Italy; ^4^ Faculty of Education, Free University of Bozen-Bolzano, Bolzano, Italy; ^5^ Clinical Nutrition Unit, Department of Endocrine and Metabolic Medicine, IRCCS Istituto Auxologico Italiano, Milan, Italy

**Keywords:** physical activity, sleep behavior, overweight, obesity, BMI, moderating effect

## Abstract

Inadequate sleep quality is a significant risk factor for overweight and obesity, which in turn may predispose individuals to adverse health outcomes. The aim of the present study was to evaluate the moderating effect of physical activity on the relationship between sleep quality and BMI in adults with overweight and obesity. In the current cross-sectional study, 589 white European participants (mean age 50 ± 12.2 years; 65% women; mean BMI 31.4 ± 5.5 kg/m^2^) were recruited from the International Center for the Assessment of Nutritional Status in Italy between October 2021 and July 2022. They completed the Godin–Shephard Leisure Time Physical Activity Questionnaire and the Pittsburgh Sleep Quality Index. The significant moderation model analysis performed on the entire sample [*F*(_3, 585_) = 4.4, *p* = 0.0045, *r* = 0.15, *r*^2^ = 0.02] found a statistically significant association between sleep quality and BMI (*β* = −0.16, *p* = 0.05), between physical activity and BMI (*β* = −0.08, *p* = 0.0018), and between the interaction of sleep quality and physical activity and BMI (*β* = 0.01, *p* = 0.01), particularly for physical activity values equal or higher than 49 Leisure Score Index (*p* = 0.004). The moderation analysis revealed a significant effect of physical activity on the relationship between sleep quality and BMI; better sleep quality was associated with lower BMI in individuals with higher levels of physical activity. The present findings suggest new aspects relating to the effect of physical activity in the relationship between sleep quality and overweight/obesity. Therefore, focusing on maintaining adequate levels of physical activity may represent an effective complementary strategy.

## 1 Introduction

Obesity and overweight are important risk factors for early mortality and several chronic health conditions (1). Some organizations have classified obesity as a chronic progressive disease, distinguishing it from simply being a risk factor for other diseases (2). Lifestyle habits are important health determinants that can influence weight and body composition, and certainly represent a good strategy to prevent them (3).

Sleep is an important modifiable lifestyle factor that can affect the risk of overweight and obesity (4, 5). Its quality and duration are crucial parameters that can influence individual health both positively and negatively (6–8). Indeed, insufficient sleep and circadian misalignment are stressors to metabolic health and are associated with adverse health outcomes, including overweight and obesity (9–13). Numerous studies highlight the interrelationship between sleep and obesity, with obesity being a leading cause of sleep alterations (14), and sleep parameters (such as a short sleep duration of 5 to 6 h/night and poor sleep quality) being important risk factors for the development of overweight and obesity (15, 16).

Physical activity (PA) includes all types of activities in day-to-day life, whether professional, domestic, or leisure-related, and represents a key modifiable lifestyle factor that can affect the risk of overweight and obesity (17). PA has been widely described as beneficial for improving health in the general population (18). Evidence suggests that high levels of PA lower the risk of overweight and obesity, and other obesity-related diseases such as metabolic, cardiovascular, and musculoskeletal diseases; depression; and certain types of cancer (19). Furthermore, high levels of PA can have beneficial effects on night-time sleep (20). In addition to PA, a more active daily routine and increased daily activity levels have been associated with better sleep quality (21).

Additionally, the relationship between sleep quality and PA has been proposed to be bidirectional (22), as they influence each other through complex and bilateral interactions (18). The intensity, volume, timing, and even the nature of the exercise may affect sleep quantity, quality, and architecture (23). Additionally, shorter sleep duration and higher daytime sleepiness may reduce PA levels leading to unfavorable conditions, ultimately reaching a morbid endpoint (24) associated with poor health outcomes (22).

Based on the current evidence, many studies have proposed the concomitant assessment of PA and sleep behavior (18, 22, 24–27), but none of them have examined whether PA could act as a moderating factor in the relationship between sleep quality and body mass index (BMI).

The present study aims to evaluate the moderating effect of PA on the interaction between sleep quality and BMI in a sample of adults with overweight and obesity.

## 2 Materials and methods

### 2.1 Participants

This cross-sectional study involved a total of 744 participants. The final sample included 589 White European participants (209 men, 35%; 380 women, 65%), who spontaneously attended the International Center for the Assessment of Nutritional Status (ICANS) in Italy for healthcare reasons between October 2021 and July 2022.

The inclusion criteria were age ≥18 years, BMI ≥25 kg/m^2^, and having filled out the two questionnaires. All the exclusion criteria were taken from medical records and included pregnancy or nursing; conditions severely limiting PA; significant cardiovascular, neurological, endocrine, and psychiatric disorders; diagnosis of obstructive sleep apnea; and any kind of medication that could potentially interfere with sleep quality.

After receiving a full explanation of the protocol, participants provided written informed consent to participate in the study. They were free to withdraw from the study at any time. The study complied with the principles established by the Declaration of Helsinki, and the Ethical Committee of the local University (n. 6/2019) approved the study procedures.

### 2.2 Study design

During the clinical visit, height and weight were measured accurately without shoes and heavy clothes. BMI was calculated as body weight in kilograms divided by the square of height in meters (kg/m^2^). Additionally, each participant filled out a form with demographic information, including whether they lived in an urban or rural environment and their educational level (years). Health status variables, such as smoking habit (yes/no), were also recorded in order to better characterize the sample but were not considered in these analyses. Furthermore, participants completed two validated questionnaires assessing PA and sleep quality, which represented the main outcomes of the study.

### 2.3 Questionnaires

#### 2.3.1 Physical activity levels

The Godin–Shephard Leisure Time Physical Activity Questionnaire (GSL-TPAQ) assessed PA. It is composed of three questions about the frequency of PA at three different intensities (mild, moderate, and strenuous) in a typical 7-day period. Each answer was multiplied by a corresponding metabolic equivalent of task value (METs 3, 5, and 9 for mild, moderate, and strenuous intensity, respectively) and summed to obtain a Leisure Score Index (LSI). A score equal to or higher than 24 classifies subjects as active, while a score lower than 23 as moderately active. Finally, a score lower than 13 corresponded to an inactive status (28).

In the current study, the moderation analysis identified three cut-off values, i.e., 14 LSI, 28 LSI, and 49 LSI, which represent the 16th, 50th, and 84th percentiles of the sample, respectively.

#### 2.3.2 Sleep behavior

The Italian version of the Pittsburgh Sleep Quality Index (PSQI) was used to evaluate sleep quality during the 30 days before compilation (29). It consists of 18 items, which evaluate different aspects of sleep. The total score ranges between 0 and 21, differentiating good sleepers (0–5) from bad sleepers (6–21) (30). The lower the final score, the better the quality of sleep.

### 2.4 Statistical analysis

The statistical analyses were carried out using the IBM Statistical Package for the Social Sciences (SPSS) Statistics (version 28; IBM Corp. Released 2019. IBM SPSS Statistics for Windows, Armonk, NY: IBM Corp) and the PROCESS macro software (macro version 4.1 for SPSS by Andrew F. Hayes) to perform the moderation analysis (31). The significance was set at *α* = 0.05 and the confidence intervals (CIs) at 95%. Data is presented as means and standard deviations (SDs), and numbers (*n*) and percentages (%) for the continuous and categorical variables, respectively. Sex (due to differences in the male and female samples) and age (due to the large range of 18–75 years) were used as covariate factors.

**Figure 1:**
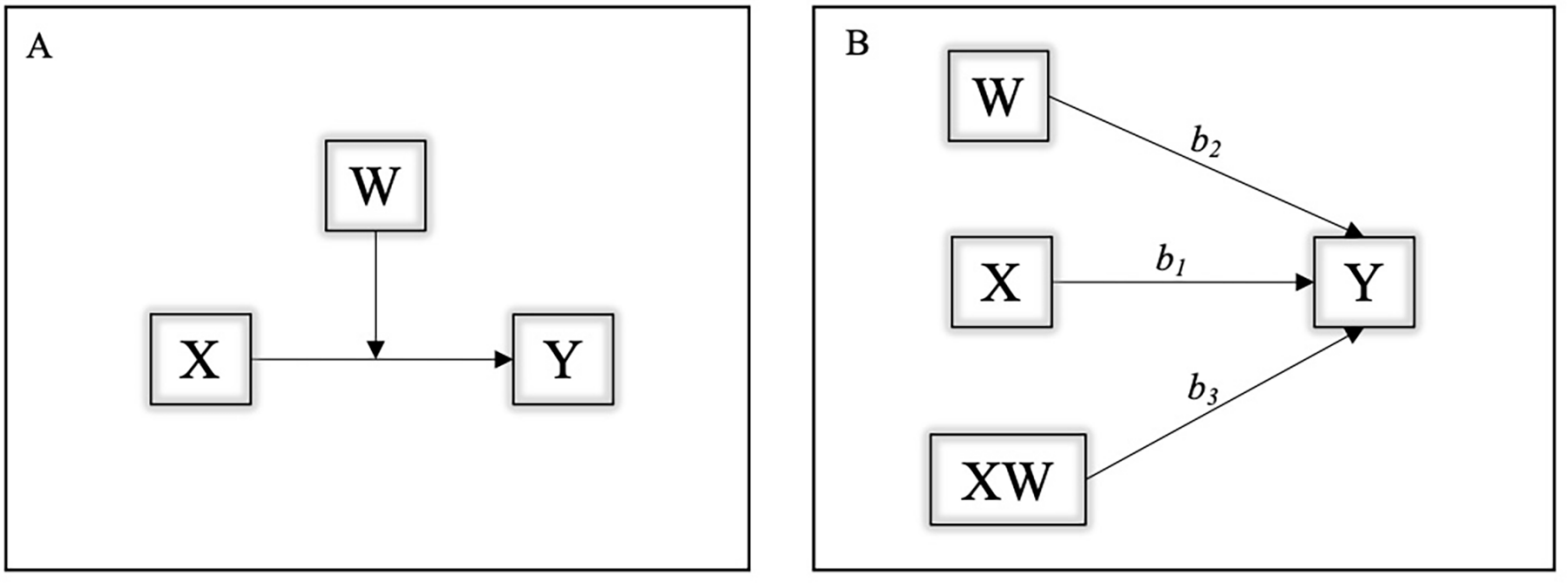
**(A,B)** Graphical representation of the moderation model. Y, dependent variable (BMI); X, independent variable (sleep); W, moderator factor (PA); *b*1, effect of the independent variable (sleep) on the dependent variable (BMI); *b*2, effect of the moderator (PA) on the dependent variable (BMI); *b*3, combined effect of the moderator (PA) and the independent variable (sleep) on the dependent variable (BMI).

Model 1 in PROCESS macro was used to verify the PA moderation role (GSL-TPAQ final score-LSI; moderator) in the relationship between sleep quality (PSQI score; independent variable) and BMI (dependent variable) (Figure 1A). Three regression coefficients describe the model: *b*1 estimates the effect of the independent variable (sleep) on the dependent variable(BMI); *b*2 estimates the effect of the moderator (PA) on the dependent variable(BMI); *b*3 estimates the effect of the interaction between the moderator (PA) and the independent variable (sleep) on the dependent variable (BMI) (Figure 1B).

We set the bootstrap number at 5,000, the conditional effects at the 16th, 50th, and 84th percentiles of the moderator's values, and all the continuous variables were centered. The Johnson–Neyman approach was used to test the cut-off point at which the GSL-TPAQ final score-LSI moderated the relationship between PSQI score and BMI. The Johnson–Neyman technique determined, along a continuum of moderator values (GSL-TPAQ final score-LSI), the region of significance in the relationship between the independent (sleep) and dependent (BMI) variables (31).

## 3 Results

### 3.1 Descriptive data

The mean age of the sample was 50 ± 12.2 years, with a mean BMI of 31.4 ± 5.5 kg/m^2^. The remaining characteristics of the sample are reported in Table 1. None of the participants took medications. Furthermore, 16% of the sample were classified as inactive (*n* = 93), 20% as moderately active (*n* = 119), 64% as active (*n* = 377), 46% as bad sleepers (*n* = 274), and 54% as good sleepers (*n* = 315).

**Table 1:** Descriptive statistics of the study sample.

Variable	Mean ± SD	*N*, %
Female		380, 65
Male		209, 35
Age (years)	50 ± 12.2	
Weight (kg)	87 ± 17.3	
Height (cm)	166.5 ± 9.2	
BMI (kg/m^2^)	31.4 ± 5.5	
GSL-TPAQ final score-LSI	31.5 ± 17.7	
GSL-TPAQ strenuous intensity (METs/week)	5.9 ± 10.7	
GSL-TPAQ moderate intensity (METs/week)	10.7 ± 10	
GSL-TPAQ mild intensity (METs/week)	15 ± 10.5	
PSQI score	5.7 ± 3.2	
PSQI-sleep quality (a.u.)	1.22 ± 0.73	
PSQI-sleep latency (a.u.)	0.86 ± 0.64	
PSQI-sleep duration (a.u.)	1.32 ± 0.99	
PSQI-sleep efficacy (a.u.)	0.59 ± 0.33	
PSQI-sleep disturbances (a.u.)	1.17 ± 0.48	
PSQI-use of sleep medications (a.u.)	0.24 ± 0.16	
PSQI-daily dysfunctions (a.u.)	0.59 ± 0.36	
Time in bed (hh:mm)	07:34 ± 01:08	
Slept hours (hh:mm)	06:30 ± 01:12	
Sleep efficiency (%)	86.23 ± 12.26	

Data were expressed as mean and standard deviation (mean ± SD) and numbers (*N*) and percentage (%).

### 3.2 Moderation analysis

**Figure 2:**
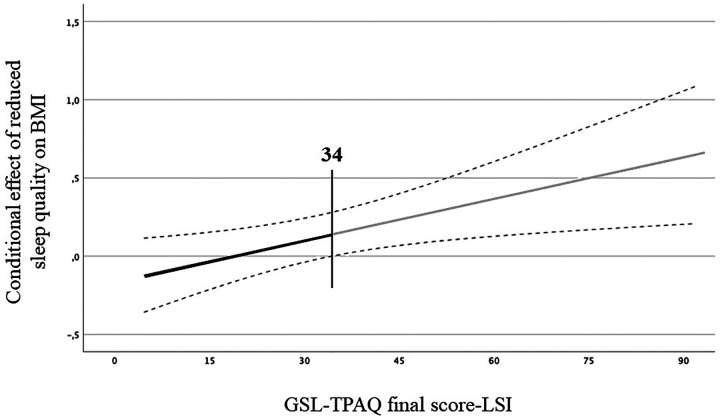
Moderation model. Regression slope for the effect of the moderator variable (GSL-TPAQ final score-LSI) on the association between sleep quality (PSQI score) and BMI, based on the Johnson–Neyman procedure. Grey line indicates the region of significance at the moderator value (34 LSI). *Statistical significance. The black line represents the neutral region of significance.

The moderation analysis results are represented in Figure 2. The model was statistically significant [*F*(_3, 585_) = 4.4, *p* = 0.0045, *r* = 0.15, *r*^2^ = 0.02]. Furthermore, the two conditional effects, *b1* (*β* = −0.16, *p* = 0.05) and *b2* (*β* = −0.08, *p* = 0.0018), were significant. The interaction between PSQI score and GLS-TPAQ final score (*b*3) was also significant (*β* = 0.01, *p* = 0.01), indicating that the association between sleep quality and BMI was moderated by PA. More specifically, the moderation of PA was significant at the 84th percentile (GLS-TPAQ value = 49 LSI; effect = 0.27, *p* = 0.004), indicating that the association between sleep quality and BMI may be moderated by PA only in those participants whose PA practice was equal or higher than 49 LSI. The test was not statistically significant at the 16th (GLS-TPAQ value = 14 LSI; effect = −0.04, *p* = 0.69) and 50th (GLS-TPAQ value = 28 LSI; effect = 0.08, *p* = 0.24) percentiles. Finally, the Johnson–Neyman test was significant at LSI values over 34, with 39.73% of cases falling above this value. This last result confirms that the moderating effect of PA on the association between sleep quality and BMI was particularly evident at the high PA level (32). In Figure 2, the slope shows the continuum of the moderator (GSL-TPAQ final score-LSI) and the region of significance. The significant region was found from 34 LSI (grey line), indicating that the negative association between reduced sleep quality, based on PSQI score, and BMI could be ameliorated for those who were above this point. A black region was observed, indicating that the effect was not effective in those with a GSL-TPAQ final score-LSI lower than the threshold.

## 4 Discussion

This study aimed to assess the moderating effect of PA on the relationship between sleep quality and BMI in a sample of 589 adults with overweight and obesity. The main finding of the current study indicates that the association between sleep quality and BMI can be moderated by PA; better sleep quality was associated with lower BMI in individuals with higher levels of physical activity. In detail, in our sample, the negative association between reduced sleep quality (based on PSQI score) and BMI could be ameliorated for those having a PA level above 34 LSI. In practical terms, this result corresponds to a mild PA regimen of at least 12 times per week for at least 15 min (or approximately 7 and 4 times per week for at least 15 min for moderate and strenuous PA, respectively). Our PA threshold for a beneficial effect on the sleep-BMI association exceeds the 24 LSI cut-off from the GSL-TPAQ, aligning with WHO guidelines recommending increased PA (33). Thus, engaging in regular and higher levels of PA appears to improve overall health and well-being, help manage overweight and obesity, and reduce the risk of obesity-related diseases (19). Increasing or maintaining PA levels over the minimum recommended by public health agencies is feasible and associated with improvements in long-term weight maintenance and overall health benefits (34, 35).

Additionally, it is important to note that PA may not be the only factor influencing the association between sleep quality and health conditions, such as maintaining a normal BMI; other factors, for example, hormonal imbalances, may also play an important role (36, 37). Anyhow, in our sample of adults with overweight and obesity, the moderation analysis underlines the beneficial effects of PA on BMI, even in individuals with poor sleep quality.

Sleep deprivation is responsible for an imbalance in the hormones that regulate appetite, such as leptin and ghrelin (7, 38). This disruption in the balance of these metabolic hormones contributes to overeating and an increased BMI (39). Additionally, insufficient sleep can lead to elevated cortisol levels, which are positively associated with food intake (40, 41). Furthermore, cortisol inhibits the appetite-suppressing effects of leptin (42) and raises plasma ghrelin levels (43, 44).

PA practice may serve as a moderator in the association between sleep and BMI through its effects on sleep, which consequently lead to an improvement in body composition. Indeed, PA can alleviate certain sleep-related symptoms (e.g., reducing sleep latency), thereby facilitating sleep onset (45, 46), which in turn reduces the propensity to eat and snack (47), finally resulting in weight loss. Furthermore, PA increases energy expenditure compared to one’s basal metabolism, which leads to a higher need for sleep recovery (23) and improvements in body composition (1, 48). By influencing sleep quality and duration, PA may also help restore the balance of leptin, ghrelin, and cortisol, ultimately contributing to changes in body composition (7, 38).

To our knowledge, this is the first investigation assessing the moderating effect of PA on the relationship between sleep quality and BMI in adults with overweight and obesity.

These results should be contextualized in view of the study’s limitations and strengths. Limitations may include the use of questionnaires instead of objective assessment of PA and sleep quality. Other limitations are the lack of parameters that better describe the body composition of our sample, the lack of other variables that could potentially influence the relationship under investigation, and the lack of mechanisms explaining how PA could moderate the association between sleep quality and BMI.

Finally, while PA appears to moderate the association between sleep quality and BMI in adults with overweight and obesity, this finding may not be generalizable to all individuals with these conditions. Our sample consists of a relatively healthy subgroup that may not represent the broader population with overweight and obesity. Despite these limitations, our preliminary results highlight a potentially valuable strategy for managing overweight and obesity: staying physically active may help control BMI, even in those with poor sleep quality.

## Data Availability

The raw data supporting the conclusions of this article will be made available by the authors, without undue reservation.
